# A case report and literature review of self-improving collodion baby in the newborn

**DOI:** 10.1097/MD.0000000000042045

**Published:** 2025-04-04

**Authors:** Yanping Guo, Zhihao Xiao, Xiaoyan Hu, Ying Liu, Guobing Chen

**Affiliations:** a Department of Pediatrics, Peking University Shenzhen Hospital, Shenzhen, China.

**Keywords:** autosomal recessive congenital ichthyosis, collodion baby, newborn, self-improving collodion ichthyosis, whole exome sequencing

## Abstract

**Rationale:**

Self-improving collodion baby (SICB) is a rare subtype of autosomal recessive congenital ichthyosis with distinct clinical features and generally favorable prognosis. This study aims to enhance understanding of SICB by examining its clinical characteristics and recent developments in diagnosis and management. Our findings provide insights that may aid in the etiological diagnosis and treatment of congenital ichthyosis.

**Patient concerns:**

We present a case of SICB treated at Peking University Shenzhen Hospital, characterized by the appearance of a collodion membrane at birth. The primary approach involved intensive moisturizing care to manage skin abnormalities.

**Diagnoses:**

Based on clinical examination and genetic testing, a diagnosis of SICB was confirmed, with mutations identified in genes commonly associated with autosomal recessive congenital ichthyosis, such as *ALOX12B*, *TGM1*, *ALOXE3*, *CYP4F22*, and *PNPLA1*.

**Outcomes:**

The patient showed significant improvement following targeted supportive care, consistent with the generally positive prognosis for SICB.

**Lessons:**

A comprehensive literature review of 31 SICB cases from 18 studies highlighted that the condition typically presents at birth with a collodion membrane. Intensive moisturizing is the main treatment, and early genetic testing is recommended to facilitate timely diagnosis and intervention. Early diagnosis can support effective genetic counseling and improve outcomes for newborns with ichthyosis.

## 1. Introduction

Self-improving collodion ichthyosis (SICI) is a rare subtype of autosomal recessive congenital ichthyosis (ARCI), characterized by the presence of typical collodion membrane features at birth, with almost complete resolution of scaling within 3 months to 1 year.^[1,2]^ At birth, infants with SICI are encased in a thick, tight, smooth, and shiny shell, formed by an overgrowth of the stratum corneum that resembles a collodion-like membrane.^[3]^

This hardened shell, resulting from significant thickening and tension in the skin, can cause various complications, including ectropion, lip deformities, and underdevelopment of nasal and ear cartilage.^[4]^ More severely, the tight membrane may interfere with critical neonatal functions such as sucking, breathing, and ventilation, leading to dehydration, malnutrition, hypoxia, and potentially life-threatening pulmonary infections.^[1]^

Although SICI’s clinical manifestations have been reported, its incidence and pathogenesis remain incompletely understood, likely involving multiple gene mutations.^[5]^ This study reports a typical case of neonatal SICI and systematically analyzes existing literature to enhance clinicians’ understanding of the condition. Improved recognition and knowledge of SICI can aid in the diagnosis, treatment, and management of similar cases in the future, offering valuable insights for medical professionals.

This manuscript is presented following the CARE reporting checklist.

## 2. Case presentation

### 2.1. Data of the present case

#### 2.1.1. Clinical materials

A female infant was admitted 30 minutes after birth due to “generalized edema with skin fissures.” The infant was born to a primiparous mother at 38^+3^ weeks of gestation age via spontaneous vaginal delivery, with a birth weight of 3050 g and no history of asphyxia. After birth, she was breastfed, had strong sucking ability, was mentally alert, showed no lethargy, and was not irritable or agitated. Jaundice appeared on the second day of life, and meconium was passed within 24 hours, with normal quantity. There was no history of consanguineous marriage, and the mother had regular antenatal checkups with no significant abnormalities. There was no similar family history.

All procedures in this study complied with the ethical standards of the Peking University Shenzhen Hospital Ethics Committee (Reference No. [2024] 119) and the revised Declaration of Helsinki (2013). The patient’s guardian provided written informed consent.

#### 2.1.2. Physical examination at admission

**Figure 1.**
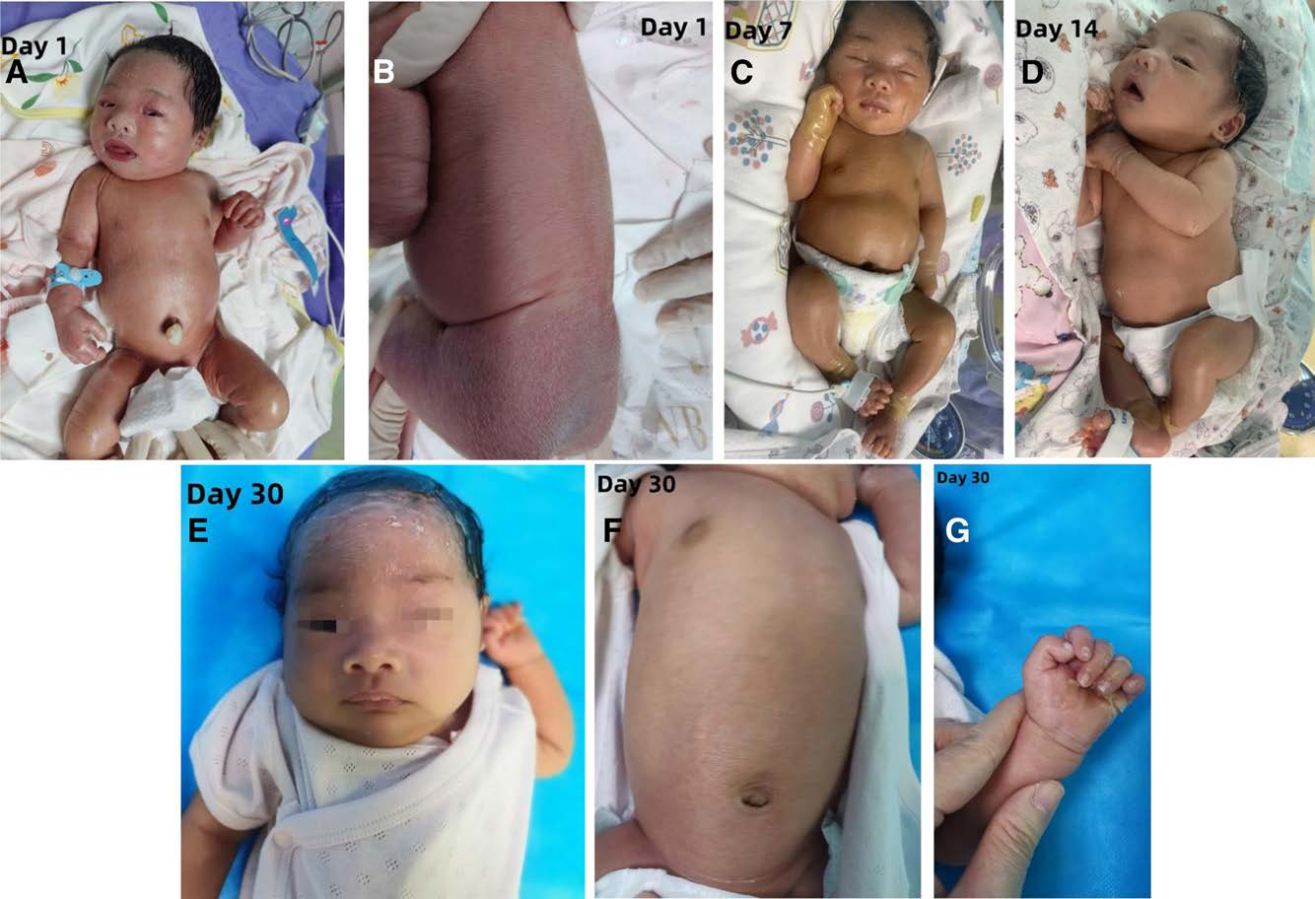
Skin evolution from birth to 30 days in this case.

Upon admission, the infant’s vital signs were as follows: temperature 36.7 °C, pulse rate 126 beats per minute, respiratory rate 55 breaths per minute, weight 3.05 kg, length 50 cm, head circumference 33.5 cm, and chest circumference 32 cm. The infant was alert with a strong cry, and the anterior fontanel was flat and soft. The skin was erythematous and shiny, covered by a tense, transparent, plastic-like membrane. Notable desquamation was observed on the limbs and buttocks, with erosions present in the groin area. The back had dry scales, both eyelids were everted, the ears were stiff, and there was swelling and fissuring of the palms. The soles were pale and edematous, and the lips were everted. Respiratory sounds were symmetrical in both lungs, without rales, and heart sounds were strong, with a regular rhythm and no murmurs. The abdomen was soft, with normal limb muscle tone and a capillary refill time of <2 seconds (see Fig. 1A and B).

#### 2.1.3. Laboratory examinations

After admission, test results indicated normal levels of myocardial enzymes, liver and kidney function, electrolytes, complete blood count, C-reactive protein, and procalcitonin. Torch IgM antibodies were negative, and both blood glucose and blood gas levels were within normal ranges. Ultrasound examinations of the brain, liver, gallbladder, and spleen showed no abnormalities. Cardiac ultrasound revealed a 2.4 mm oblique fissure in the middle of the atrial septum, and a 2 to 3 mm discontinuity near the apex of the interventricular septum, with no pericardial effusion observed. X-rays of the hands and feet showed mild soft tissue swelling in both hands, with no abnormalities in the bones of the feet. The patient’s 25-hydroxyvitamin D level was 16.15 ng/mL, G6PD activity was normal, and blood ammonia was 46.6 μmol/L.

#### 2.1.4. Diagnosis and treatment

##### 2.1.4.1. Diagnosis: SICI

Treatment: the infant was placed in a humidified incubator with continuous monitoring of vital signs to prevent fluid and electrolyte imbalances. Intensive skin moisturizing care was provided by applying a mixture of petroleum jelly and medical moisturizing cream every hour to the entire body to prevent scaling and secondary infections. Erythromycin ointment was applied to the areas of erosion, and erythromycin eye ointment was applied every night to prevent eye infections. Both the infant and the parents underwent multi-gene sequencing for hereditary skin disorders. Oral administration of vitamin A (2000 IU) and vitamin D (700 IU) was initiated, and skin care continued throughout the treatment. One week after birth, the infant began to shed skin (Fig. 1C), with peeling mostly completed on the face and trunk by the second week (Fig. 1D). After 15 days of treatment, the infant’s condition stabilized, and by discharge, the skin had returned to normal, except for the head and extremities (Fig. 1E–G).

#### 2.1.5. Results of gene sequencing

**Figure 2.**
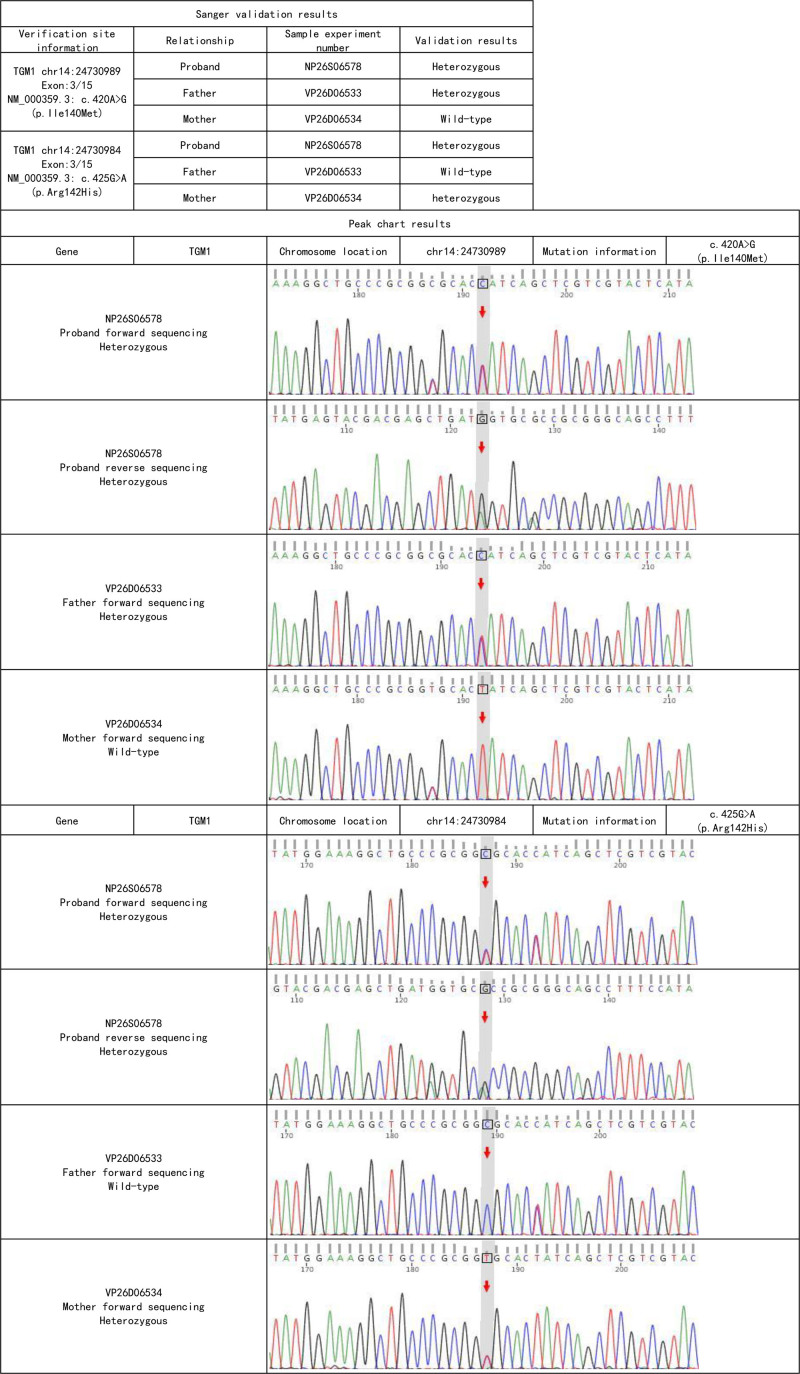
Sanger sequencing chart.

The proband and her parents underwent whole exome sequencing of anticoagulated peripheral venous blood, followed by Sanger sequencing to validate the suspected pathogenic variants. According to the ACMG classification guidelines for genetic mutations, the patient was identified as having compound heterozygous pathogenic variants in the *TGM1* gene (see Table 1). The patient carries the c.425G > A (p. Arg142His) variant inherited from her mother, which has been reported to reduce TGase activity (PMID:19278426) and is classified as a likely pathogenic variant. The patient also carries the c.420A > G (p. Ile140Met) variant inherited from her father, which has been previously observed in compound heterozygous ichthyosis patients and is also classified as a likely pathogenic variant (see Fig. 2).

**Table 1:** Pathogenic or likely pathogenic single nucleotide variants (SNVs) and small insertion/deletion variants (Indels) in the WES results that can explain the phenotype of the examined individual.

Gene	Chromosomal location	Variant information	Zygosity type	Disease name	Inheritance pattern	Variant origin	Variant classification
TGM1	chr14:24730984	NM 000359.3:c.425G > A(p.Arg142His)	Heterozygous	ARCI type 1 [MIM:242300]	AR	Mother	Suspected pathogenic variant
TGM1	chr14:24730989	NM 000359.3:c.420A > G(p.lle140Met)	Heterozygous	ARCI type 1 [MIM:242300]	AR	Father	Suspected pathogenic variant

A = adenine, AR = autosomal recessive, ARCI = autosomal recessive congenital ichthyosis type 1, Arg = arginine, G = guanine, His = histidine, Ile = isoleucine, Met = methionine, NM = refers to the RefSeq (NCBI Reference Sequence) accession number for a specific mRNA transcript.

#### 2.1.6. Literature review

**Figure 3.**
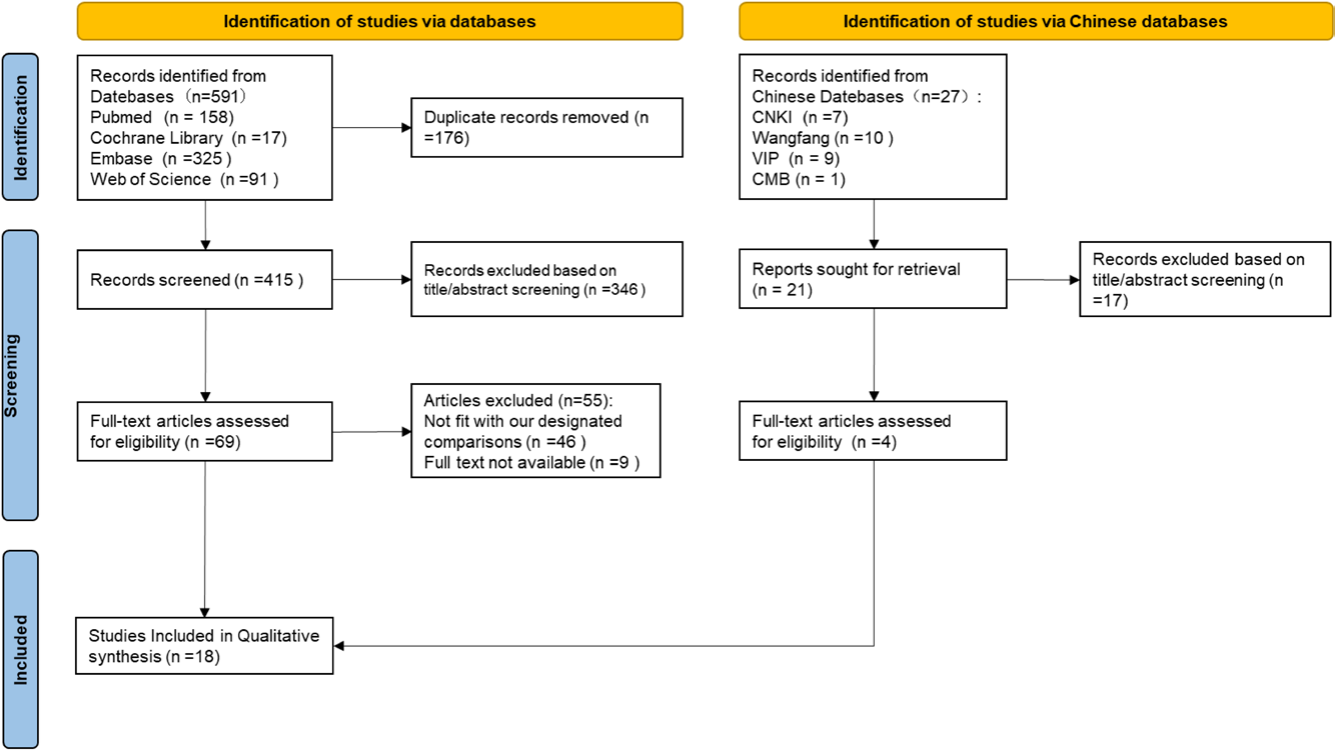
Flow chart for the selection of studies.

A literature search was conducted using the keywords “collodion baby,” “self-improving collodion ichthyosis,” “autosomal recessive congenital ichthyosis,” and “neonates” in databases including CNKI, Wanfang Database, VIP Database, Chinese Medical Journal Full-Text Database, PubMed, Embase, Web of Science, and Cochrane Library, with data up to July 27, 2024. The search focused on studies involving neonates diagnosed with self-improving collodion ichthyosis and with genetic testing results, excluding cases with additional diseases. All identified records were downloaded to Endnote X9, and duplicates were removed both automatically and manually. Two independent researchers screened the studies by title and abstract, selected eligible cases, and obtained full texts for further review. From the 618 publications identified, 436 articles remained after removing duplicates. Of these, 363 were excluded based on title and abstract evaluation. Among the remaining 73 studies, 55 did not meet the inclusion criteria, and 9 were excluded due to the unavailability of full texts. Ultimately, 18 studies^[6–23]^ were included, as shown in Figure 3 and Table S1, Supplemental Digital Content, http://links.lww.com/MD/O608. Of these, 14 were in English and 4 in Chinese. After removing duplicate cases, a total of 31 patients were analyzed.

The clinical manifestations of self-improving collodion ichthyosis in neonates were summarized. Among the 31 patients, 16 were female (51.6%), and 22 were full-term infants (71.0%). At birth, all infants were covered with a thick, varying-layer collodion membrane. Symptomatic supportive treatments, including localized or systemic skin moisturizing and infection prevention, were applied. The membrane naturally peeled off within 2 to 4 weeks, revealing near-normal skin, and most patients had nearly normal skin by 2 to 4 months, with only a few showing mild ichthyosis characteristics later. Genetic analysis revealed common mutations including *ALOX12B* (51.61%, 16/31), *TGM1* (22.58%, 7/31), *ALOXE3* (12.90%, 4/31), *CYP4F22* (9.68%, 3/31), and *PNPLA1* (3.23%, 1/31). The clinical prognosis was generally good, consistent with the commonly reported symptoms of self-improving collodion ichthyosis in the literature (see Table 2).

**Table 2:** Characteristics of studies included in the literature review.

Author (year)	Number of cases (n)	Sex	Full-term/premature	Postnatal clinical manifestations	Treatment	Mutated gene	Prognosis
Hao (2024) ^[6]^	1	F	Full-term	Collodion baby, eyelid eversion, diffuse skin scaling with erythema	ABC	TGM1	b
Chen (2024) ^[7]^	1	M	Premature	Diffuse skin scaling with erythema	ABC	ALOXE3	b
Yu (2023) ^[8]^	1	M	Full-term	Collodion baby, eyelid eversion	ABC	ALOX12B	a
Amoedo (2023) ^[9]^	1	M	Full-term	Collodion baby, eyelid edema and eversion	ABC	ALOX12B	b
Zhu (2023) ^[10]^	1	F	Full-term	Collodion baby	ABC	ALOX12B	a
Li (2022) ^[11]^	1	F	Premature	Collodion baby, eyelid eversion, syndactyly	ABC	PNPLA1	a
Zhang (2022) ^[12]^	1	M	Full-term	Collodion baby, erythroderma with multiple fissures on the skin, eyelid edema and eversion, deformities of the external ear and auricle	ABC	ALOX12B	a
Takeichi (2022) ^[13]^	1	M	Full-term	Collodion baby, moderate fissures at the joints	ABC	CYP4F22	b
Yang (2021) ^[14]^	3	2 M, 1 F	2 Full-term, 1 premature	Collodion baby, eyelid eversion, auricular forward folding, limb swelling with restricted movement	ABC	ALOX12B	b
Noguera-Morel (2016) ^[15]^	2	2 M, 1 F	1 Full-term, 1 premature	Collodion baby	ABC	CYP4F22	b
Santesteban (2016) ^[16]^	1	M	Premature	Collodion baby, eyelid eversion, nail deformities	ABC	ALOX12B	b
Tanahashi (2014) ^[17]^	1	F	Full-term	Collodion baby	ABC	TGM1	a
Bourrat (2012) ^[18]^	1	M	Full-term	Collodion baby	ABC	TGM1	a
Theiler (2010) ^[19]^	1	F	Full-term	Collodion baby	ABC	TGM1	a
Vahlquist (2010) ^[20]^	9	2 M, 7 F	Premature	Collodion baby	ABC	6ALOX12B, 3 ALOXE3	b
Mazereeuw-Hautier (2009) ^[21]^	1	F	7 Full-term, 2 premature	Collodion baby	ABC	TGM1	a
Harting (2008) ^[22]^	2	M	Premature	Collodion baby	ABC	ALOX12B	a
Raghunath (2003) ^[23]^	2	F	Full-term	Collodion baby	ABC	TGM1	a

A: symptomatic support; B: skin moisturizing; C: infection prevention; a: skin completely normal; b: localized skin has a few thin scales.

## 3. Discussion

This study systematically reviews and summarizes the genetic mutations associated with SICI, revealing that mutations in the *ALOX12B* gene are a major cause of this transient ARCI, accounting for 51.61% of the 31 genotyped cases. These findings are consistent with previous research. The *ALOX12B* gene encodes 12R-lipoxygenase, an enzyme essential for skin barrier formation and keratinocyte differentiation. Mutations in the *ALOX12B* gene impair lipid metabolism, leading to skin barrier dysfunction and clinical manifestations characteristic of collodion baby in neonates.^[24]^

Additionally, mutations in the *TGM1* gene are identified as the second most common cause of SICI. The *TGM1* gene encodes an enzyme crucial for the terminal differentiation of skin keratinocytes, catalyzing the cross-linking reactions of keratins to form a robust extracellular matrix that maintains skin barrier integrity.^[25]^ Whole exome sequencing confirmed *TGM1* mutations in the patient, identifying 2 suspected pathogenic variants: c.425G > A (p. Arg142His) and c.420A > G (p. Ile140Met), inherited from the mother and father, respectively, resulting in compound heterozygous mutations. Although these mutations reduce *TGM1* enzyme activity, they do not completely eliminate its function. The p. Arg142His and p. Ile140Met mutations may impact different domains or functional regions of the enzyme. Although these mutations impair the skin barrier at birth, environmental factors and compensatory differentiation pathways in keratinocytes contribute to gradual skin improvement. At birth, the patient’s skin was covered with a tense, glossy, transparent membrane, consistent with the clinical presentation of SICI. Typically, this collodion-like membrane peels off within weeks, and the skin gradually returns to normal or exhibits only minor damage. Consequently, SICI is also referred to as self-healing collodion baby. In this case, the clinical presentation was consistent with the genetic findings, confirming the diagnosis of SICI. It is noteworthy that not all SICI patients exhibit identical locations of skin damage, which may be associated with different pathogenic gene mutations.

We also identified *ALOXE3* gene mutations as the third most common cause of SICI. The *ALOXE3* gene encodes epidermal lipoxygenase 3, which is critical for skin barrier formation by oxidizing lipids in the stratum corneum and facilitating epidermal barrier maturation. Compound heterozygous mutations in *ALOXE3* can affect the activity of epidermal lipoxygenase 3, resulting in initial defects in stratum corneum structure. However, with the turnover of stratum corneum cells and the dynamic reconstruction of the skin barrier, the skin gradually adapts to these mutations, leading to improved function.^[26]^

In this study, *CYP4F22* mutations were found to be a less common cause of SICI, accounting for 9.68% of the 31 genotyped cases. *CYP4F22* encodes a cytochrome P450 enzyme that is essential for the metabolism of fatty acids in the stratum corneum, contributing to the production of very long-chain fatty acids and keratinization. Compound heterozygous or homozygous mutations in *CYP4F22* can reduce enzyme activity, impairing lipid metabolism and resulting in a collodion-like appearance.^[27]^

Among the SICI cases in our study, only 1 patient had compound heterozygous *PNPLA1* mutations. The *PNPLA1* gene encodes a protein from the patatin-like phospholipase family, responsible for producing ceramide fatty acids, which are crucial for maintaining skin barrier integrity. *PNPLA1* mutations may alter the composition of stratum corneum lipids at birth, resulting in a collodion-like appearance.^[28]^ However, with the turnover of stratum corneum cells and the reconstruction of lipid composition, skin function gradually recovers. Spontaneous adjustments in lipid metabolism partially compensate for the defects caused by the mutation, promoting skin self-healing.

The strength of this study lies in its comprehensive literature search, which includes major English and Chinese databases, thereby minimizing the risk of omitting important published reports. To our knowledge, this is the largest study on the etiology of SICI conducted to date. The phenotypes of SICI patients vary according to different mutation types, and the precise genotype–phenotype correlation remains to be fully elucidated.

In conclusion, SICI exemplifies the genetic diversity and clinical overlap among various ARCI subtypes. Only through meticulous analysis of clinical and genetic findings can the SICI subtype of ARCI be clearly delineated. For clinicians, understanding ichthyosis cases that transition from severe to mild, along with their recessive genetic characteristics, is crucial for delivering precise genetic counseling.

## Acknowledgments

Thanks to all authors for their contributions to this study.

## Author contributions

**Conceptualization:** Yanping Guo.

**Data curation:** Yanping Guo, Zhihao Xiao, Xiaoyan Hu, Ying Liu, Guobing Chen.

**Supervision:** Guobing Chen.

**Writing – original draft:** Yanping Guo.

**Writing – review & editing:** Yanping Guo.

## Supplementary Material


