# Supplementary material for: A case report and literature review of self-improving collodion baby in the newborn
Source: Medicine (Baltimore). 2025 Apr 4;104(14):e42045. doi: 10.1097/MD.0000000000042045 (PMC11977717; doi:10.1097/MD.0000000000042045)
Supplement: Supplementary file 1 [file medi-104-e42045-s001.docx]

| **Table S1. Record of database search strategies - run 27/07/24.** | | |
| --- | --- | --- |
| **#** | **Pubmed search strategy** | **Results** |
| # 1 | (((Collodion baby[Title/Abstract]) OR (self-improving collodion ichthyosis[Title/Abstract])) OR (autosomal recessive congenital ichthyosis[Title/Abstract])) | 439 |
| # 2 | ((((breastmilk[tiab] OR (human[tiab] OR breast[tiab] OR mother*[tiab] OR maternal[tiab] OR express*[tiab] OR donor*[tiab] OR donated[tiab] OR bank*[tiab])) AND milk*[tiab])) OR ((breastfeed*[tiab] OR breastfed[tiab] OR ((breast[tiab] OR HM[tiab]) AND (fed[tiab] OR feed*[tiab]))))) OR(((EHM[tiab] OR MOM[tiab] OR PDM[tiab]) AND milk[tiab])) | 800,329 |
| # 3 | #1 AND #2 | 158 |
| **#** | **Embase strategy** | **Results** |
| # 1 | 'collodion baby'/exp OR 'collodion baby' OR (('collodion'/exp OR collodion) AND ('baby'/exp OR baby)) OR 'self-improving collodion ichthyosis':ab,ti OR 'autosomal recessive congenital ichthyosis':ab,ti | 1,681 |
| # 2 | 'infant, newborn'/exp OR 'infant, newborn' OR (('infant,'/exp OR infant,) AND ('newborn'/exp OR newborn)) OR 'infants, newborn':ab,ti OR 'newborn disease':ab,ti OR 'newborn infants':ab,ti OR neonate:ab,ti OR newborn:ab,ti | *795,815* |
| # 3 | #1 AND #2 | *325* |
|  | **Cochrane search strategy** | **Results** |
| # 1 | ("Collodion baby"):ti,ab,kw OR (self-improving collodion ichthyosis):ti,ab,kw OR (autosomal recessive congenital ichthyosis):ti,ab,kw | 17 |
| # 2 | ("Infant, Newborn"):ti,ab,kw OR (Infants, Newborn) :ti,ab,kw OR (Newborn Infant):ti,ab,kw OR (Newborn Infants) :ti,ab,kw OR (Neonate):ti,ab,kw | 45,515 |
| # 3 | ((brain OR neurologic* OR cognitive* OR intellectual* OR motor OR psychomotor) NEAR/1 development*) OR ((infan* OR child*) NEAR/1 development*) OR ((neurodevelopment*) OR (neuro-development*) OR (neurocognitive*) OR (neurocognitive) OR (neurobehavior*) OR (neurobehaviour*) OR (neuro-behavior*) OR (neuro-behaviour*) OR (neuropsycholog*) OR (neuro-psycholog*) OR cognition OR intelligence OR (executive function)) | 67,398 |
| # 4 | (#1 AND #2 AND #3)with Publication Year from 2000 to 2023, in Trials | 399 |
|  | **Web of Science search strategy** | **Results** |
| # 1 | ((TS=(Collodion baby)) OR TS=(self-improving collodion ichthyosis)) OR TS=(autosomal recessive congenital ichthyosis) | *781* |
| # 2 | ((((((TS=(Infant, Newborn)) OR TS=(Newborn Infant)) OR TS=(Newborn Infants)) OR TS=(Neonate)) OR TS=(Neonates)) OR TS=(Newborns)) OR TS=(Newborn) | 287,698 |
| # 3 | #1 AND #2 | *91* |
| **China National Knowledge Infrastructure (CNKI)** | | |
| (主题:胶棉宝宝)OR(主题:自我改善型胶棉鱼鳞病)OR(主题:常染色体隐性遗传先天性鱼鳞病)AND(主题:新生儿) | | 7 |
| **Wanfang Database (Wangfang)** | | |
| 主题:(胶棉宝宝) or 主题:(自我改善型胶棉鱼鳞病) or 主题:(常染色体隐性遗传先天性鱼鳞病) and 主题:(新生儿) | | 10 |
| **China Science and Technology Journal Database (VIP)** | | |
| ((题名或关键词=胶棉宝宝 OR 题名或关键词=自我改善型胶棉鱼鳞病) OR 题名或关键词=常染色体隐性遗传先天性鱼鳞病) AND (题名或关键词=新生儿) | | 9 |
| **China Biology Medicine disc (CBM)** | | |
| [("胶棉宝宝"[摘要:智能] OR "自我改善型胶棉鱼鳞病"[摘要:智能] OR "常染色体隐性遗传先天性鱼鳞病"[摘要:智能] )AND](javascript:toDoRelimitSearch();)  [( "新生儿"[摘要:智能])](javascript:toDoRelimitSearch();) | | 1 |
